# Post-traumatic stress, anxiety and depression after intensive care unit stay: Findings from a general hospital

**DOI:** 10.1192/j.eurpsy.2021.388

**Published:** 2021-08-13

**Authors:** U. Bhaumik, V. Subramaniyam, R. Kandukuru

**Affiliations:** 1 Psychiatry, Independent Practice, Kolkata, India; 2 Psychiatry, Independent practice, Bangalore, India; 3 Anaesthesiology And Critical Care, M S Ramaiah Medical College, Bangalore, India

**Keywords:** post-traumatic stress disorder, anxiety and depression, delusional memories, intensive care unit

## Abstract

**Introduction:**

Post-traumatic stress disorder (PTSD) following intensive care is a relatively new entity. It is triggered due to traumatic experiences in a setting of threat to life due to illness. Prolonged stay in intensive care predisposes to delusional memories related to the stay experience and may increase likelihood of post-traumatic stress.It may also present as anxiety or depression.

**Objectives:**

This study explored the prevalence of post-traumatic symptoms in intensive care, find its correlates and its impact on health-related quality of life (HRQoL).

**Methods:**

225 adult patients admitted for at least 1 day in the intensive care unit (ICU) of a general hospital in Bangalore,India were recruited and assessed at 1 week,1 month and 3 months after ICU discharge. Subjects were assessed for ICU related memories, PTSD, anxiety and depression scores and quality of life at and post discharge.

**Results:**

59.6% of the study population had significant post-traumatic stress, including anxiety in 62.35%, depression in 10.58% and mixed anxiety-depression in 27.06%. Delusional memories were found in 31.6%. Presence of delusional memories was found to have significant correlation with post-traumatic stress and had a negative impact on HRQoL.

**Conclusions:**

This study was the first of its kind from Asia. More systematic studies on PTSD following ICU stay and its correlates are required as available evidence lacks homogeneity. Suitable preventive measures should be taken to reduce prevalence of post-traumatic stress in intensive care due to its lasting impact on HRQoL.

**Disclosure:**

No significant relationships.

